# Employment Status and Work Ability in Adults with Cystic Fibrosis

**DOI:** 10.3390/ijerph182211776

**Published:** 2021-11-10

**Authors:** Veruscka Leso, Vincenzo Carnovale, Paola Iacotucci, Daniela Pacella, Rosaria Romano, Ilaria Della Volpe, Ivo Iavicoli

**Affiliations:** 1 Section of Occupational Medicine, Department of Public Health, University of Naples Federico II, Via S. Pansini 5, 80131 Naples, Italy; Veruscka.leso@unina.it (V.L.); rosaria_romano@libero.it (R.R.); ilariadellavolpe92@gmail.com (I.D.V.); 2 Adult Cystic Fibrosis Center, Department of Translational Medical Sciences, University of Naples Federico II, Via S. Pansini 5, 80131 Naples, Italy; vincenzo.carnovale@unina.it (V.C.); paola.iacotucci@unina.it (P.I.); 3 Department of Public Health, University of Naples Federico II, Via S. Pansini 5, 80131 Naples, Italy; daniela.pacella@unina.it

**Keywords:** cystic fibrosis, work ability, employment, capacity to work, occupational health, professional life, quality of life, disease management, social inclusion, career counseling

## Abstract

Improvements in the survival and clinical outcomes of cystic fibrosis (CF) patients raised questions about their workforce participation and capacity to work. One hundred and ninety-six outpatients, attending the Adult CF Center of an Italian University Hospital, were enrolled between May 2020 and March 2021. The patients’ personal and clinical characteristics, employment status, and profession were assessed. The Cystic Fibrosis Questionnaire—Revised and the work ability index (WAI) were employed to assess CF health-related quality of life and the employee’s perception of their ability to work, respectively. Among the enrolled patients, 98 (50%) were employed. The non-working subjects were significantly younger (mean age ± standard deviation: 30 ± 10 vs. 37 ± 10 years) and were diagnosed with CF significantly earlier (9 ± 13 vs. 17 ± 18 years) than the employed subjects. The vast majority of CF workers (82.6%) were employed in tertiary professions. A general good work ability perception was determined in the employed population. Aging and being employed for >15 years could significantly predict a reduction in work ability, while a better quality of life was a positive predictor for its enhancement. Although further research is necessary, these results may introduce interdisciplinary CF healthcare management that includes a work function assessment, formal career counseling, and job guidance to support the personal, social and professional lives of CF patients.

## 1. Introduction

Cystic fibrosis (CF) is an inherited disorder that causes alterations to the lungs, digestive system, and other organs in the body [1,2]. Formerly a pediatric disease, CF has reached adulthood as, in the last few decades, the development of key drugs, intensive use of antibiotics, coordinated care packages, and nutritional support has significantly improved patients’ survival and clinical outcomes [3,4,5]. Indeed, the proportion of CF adults currently exceeds 50% in most countries, and is expected to increase by 75% by 2025 [6]. This evidence supports the importance of comprehensively managing the disease in a way that takes into account the patients’ desire for married life and parenthood, as well as professional vocation and employability [7].

In the latter regard, although a generally good worldwide rate of employment has been reported for CF patients, their work ability, intended to be the interplay between the specific job characteristics and the individual’s features, has not been fully explored [8]. This seems to be relevant considering the increasing workforce participation of patients, the physical–psychological limitations induced by such a chronic and progressive disease, and the hard balance needed between work duties and the large burden of care. 

Therefore, the present study aimed to assess employment status, type of occupation performed, and work ability in a cohort of Italian CF patients, and to identify individual, pathological and occupational factors affecting labor force participation and capacity for work. Overall, this may be important to understand the impact that the physical and emotional implications of CF may have on work functioning, in order to achieve a multidisciplinary, comprehensive management of the disease, including not only clinical strategies, but also adequate support of patients’ occupational health to improve disease outcomes, and their personal, social and professional lives. 

## 2. Materials and Methods

### 2.1. Participants and Study Design

Outpatients attending the Adult CF Reference Center of the University Hospital “Federico II” in Naples, Campania region, Italy, in the period between May 2020 and March 2021, were invited to participate in this cross-sectional study. Patients ≥ 18 years were considered eligible if they had a confirmed diagnosis of CF and were able to provide written informed consent to participate in the study. Only patients unable or unwilling to provide consent were excluded from the research. The study protocol was reviewed and approved by the Ethics Committee of the University of Naples “Federico II” (n. 61/2020).

### 2.2. Data Collection

A screening form, completed by the clinical staff, was employed to obtain information about patients’ demographic and personal characteristics as summarized in Table 1. Concerning clinical data, the age at diagnosis was collected together with information on hospital admissions, respiratory symptoms and oral antibiotic or oxygen therapy within the 12 months before the survey. The results of the pulmonary function tests performed during the clinical visit (forced vital capacity (FVC), forced expiratory volume in the first second (FEV1) and the ratio FEV1/FVC) were registered both in absolute value and as percentage of predicted value. The sampling population was divided into employed and unemployed. The latter group included students, homemakers, people seeking a job and inactive persons (i.e., retired individuals and those not seeking a job). Moreover, fixed term or permanent engagement, as well as full-time or part-time schedules, were assessed. The International Standard Industrial Classification of All Economic Activities was employed to classify the productive field of employment [9]. Jobs were divided into 10 occupational activity families according to the International Standard Classification of Occupations established by the International Labour Organization [10]. According to these families, employees were grouped into “white collar”, those engaged in skilled jobs, requiring a certain formal education, and “blue collar”, those involved in unskilled, manual jobs, not requiring specific training. Occupational risks experienced during specific job tasks, occupational health surveillance programs adopted in the workplace and the patients’ fitness for work were also explored.

### 2.3. Health-Related Quality of Life

The Cystic Fibrosis Questionnaire–Revised (CFQ–R) was employed as a validated and internationally established specific instrument to assess health-related quality of life in CF [11]. This self-reported questionnaire comprises 50 questions that assess the general domains of vitality, emotional, health perception, physical functioning, and role functioning, and CF-specific domains of body image, eating, treatment burden, respiratory and digestive symptoms [12]. The CFQ–R relies on 2–week recall and is based on a four-point Likert response scale. Total scores range from 0 to 100, with higher scores being indicative of a better quality of life [13].

### 2.4. Work Ability Assessment

The employee’s perception of work ability was assessed through the self-administered work ability index (WAI) [14,15]. This is a summary measure of seven items including the following: individuals’ current ability to work in comparison with their best years of life; their ability to work concerning their demand for work; the number of diagnosed diseases or limitations from which they suffer; their estimated impairments due to diseases/abilities or limitations; the number of sick leaves they have taken during the previous year; self-prognosis of work ability for the next 2 years. The WAI score ranges from 7 to 49 points. Points of 7–27, 28–36, 37–43, and 44–49 correspond to low, moderate, good and excellent work ability, respectively [16]. 

### 2.5. Statistical Methods

Data are presented as frequency (percentages) for the categorical variables, while they are presented as mean ± standard deviation for continuous variables. χ^2^ test and Fisher’s test as appropriate were used to test the differences among the groups for categorical variables. Student’s *t*-test or Mann–Whitney U as appropriate were used to test the differences among the groups for continuous variables. A linear regression model was used to predict WAI score using the reported variables, while logistic regression model was used to investigate the predictors of employment status. Estimates of the logistic regression coefficients are exponentiated and reported as odds ratios (OR). The significance level for all analyses was set to α = 0.05. All analyses were performed using the statistical software R (version 4.0.3) [17].

## 3. Results

### 3.1. Characteristics of Study Participants

A total of 240 patients were asked to participate. Of these, 196 (81.6%) subjects agreed to be enrolled. The main characteristics of the study participants are listed in Table 1. Overall, a total of 104 (53%) patients were male, and the mean age ± standard deviation (SD) of the participants was 33.4 ± 10.6 years. The average BMI ± SD was 24.2 ± 4.2. Eighty-two percent of the population were non-smokers. Concerning educational levels, 38 patients (19%) only attended primary or secondary school, 113 (58%) had at least a high school diploma, and 45 (23%) had a degree or post-graduate degree. The majority of the patients were single (58%). From a clinical perspective, the mean age at CF diagnosis was 12.7 ± 16.2 years. Almost all of the enrolled patients did not receive intravenous antibiotics (85%), oxygen therapy (92%), or hospital admission (91%) during the previous 12 months. The mean ± SD percentages of the predicted values for the spirometric parameters were as follows: 76 ± 30%, 86 ± 23%, and 72 ± 13% for FEV1, FVC, and FEV1/FVC, respectively. 

### 3.2. Employment Status and Predictive Factors

Among the total enrolled population, 98 (50%) patients were employed outside of their home at the time of the survey. The length of employment was 1–5 years in 36 (37%) of the CF workers, followed by 20 (20%) who were employed for less than 1 year, and 16 (16%), 9 (9%), and 17 (17%) who were employed for 6–10, 11–15, and >15 years, respectively. Among the remaining 98 unemployed subjects, 29 (30%) were students, 26 (27%) were homemakers, 29 (30%) referred to be looking for a job, while 14 (14%) reported to not be seeking employment at all. In only five cases, inactivity was attributed to CF. The characteristics of the working and non-working groups are summarized in Table 1.

The non-working patients were significantly younger than the employed patients at the time of the survey, and they were also diagnosed with CF significantly earlier. Concerning gender distribution, a significantly greater percentage of males were employed, while, regarding marital status, a significantly higher portion of single individuals were present among the unemployed patients. Although, in both groups, generally high educational levels were reported, a significantly different distribution emerged. In fact, a higher percentage of subjects with a degree or post-graduate degree (31%) was represented in the working group compared to the non-working group (15%), while, in the latter, a greater portion obtained a high school degree (63% vs. 52%, respectively). In regards to the quality of life, the employed subjects reported significantly better scores in specific CFQR sub-domains, such as physical, role functioning, treatment burden, and body image. With respect to the clinical features, the only significant difference was determined for the mean FVC level expressed in liters, which, although it remained in a normal range, was significantly higher in workers compared to non-working patients. 

According to the above-described differences, the logistic regression analysis (Table S1) confirmed aging (OR 1.07, 95% CI [1.04–1.11], *p* = <0.001) and male gender (OR 2.73, 95% CI [1.54–4.91], *p* = <0.001) as significantly associated with an increased possibility of being employed. Furthermore, a higher educational level was positively associated with employment, as subjects with a degree or post-graduate degree were more than twice as likely to be in the workforce compared to those with only primary or secondary education (OR 2.47, 95% CI [1.02; 6.13], *p* = 0.037). Concerning clinical parameters, the possibility of workforce participation was significantly enhanced according to the increase in the levels of FEV1 L (OR 1.27, 95% CI [1.00–1.63], *p* = 0.05) and FVC L (OR 1.38, 95% CI [1.09–1.75], *p* = 0.006), while no significant associations emerged with hospital admissions, intravenous antibiotics, or oxygen-based therapies. In regards to the quality of life, better scores in physical (OR 1.01, 95% CI [1.00–1.02], *p* = 0.035) and role functioning (OR 1.02, 95% CI [1.00–1.03], *p* = 0.017), as well as body perception (OR 1.02, 95% CI [1.00–1.03, *p* = 0.009) and treatment burden (OR 1.02, 95% CI [1.00–1.03], *p* = 0.009), were significantly associated with a greater possibility of being engaged in an occupation. 

### 3.3. Job Characteristics

Concerning job schedule, 65 (66%) and 33 (34%) of the workers were involved in full- and part-time jobs, respectively. However, no differences in terms of demographic, personal and clinical features emerged between the two groups, with the exception of a greater portion of males employed full-time, a different distribution in sport activities, and a greater presence of digestive symptoms in this group (*p* < 0.05) (Table 2). Half of the working population (n. 51, 52%) reported having a permanent contract, 25 (25%) had a fixed-term contract, while 22 (22%) failed to provide details in this regard. The job sectors in which CF patients were engaged in are summarized in Table 3. The wholesale and retail trade (16%), as well as the healthcare (13%) sectors, were the most represented fields of activity. The vast majority of CF workers (n. 81, 82.6%) were employed in white-collar professions, including managerial, professional and technical tasks, clerical, service and sale occupations. The remaining portion was engaged in blue-collar activities, as laborers, craftsmen, farmers, drivers, and armed forces employees.

When patients were asked about the occupational risks experienced during the activities in their job, they primarily referred to the use of video terminal displays (n. 19; 19%); the adopted postures (n. 14; 14%); biological (n. 12; 12%) and chemical (n. 11; 11%) risk factors; manual handling of loads (n. 8; 8%); shift or night-shift work (n. 9; 9%). Lower percentages of workers reported noise (n. 6; 6%) and vibration exposure (n. 6; 6%), as well as the handling of low loads at high frequency (n. 6; 6%). 

Half of the CF workers (52%) were subjected to routine workplace health surveillance programs, and only a limited percentage (2%) of them requested an extraordinary medical examination due to difficulties related to working with CF. Concerning the fitness for work, the vast majority of the enrolled patients (76%) were judged as fit for their job tasks, while 10 (20%) received some prescriptions to follow while performing their work, or had limitations in their job tasks, including a reduction in the hours spent at work, and limitations in the use of specific machinery or in physical workload, and 4 (8%) received a judgment of unfitness to perform their tasks. Sixty-six workers (66%) reveled their disease in the workplace, and in 39 (59%) cases, the recipients of this confidence were their colleagues.

### 3.4. Work Ability and Predictive Factors 

A general good work ability level was determined in the employed population, as the respondents’ WAIs stood, on average ± SD, at 38.9 ± 5.1 points (Table 1). Categorization of the WAI scores showed that 22% and 52% of the CF workers had a good or excellent work ability, respectively, while a more limited portion of patients reported moderate (22%) or poor (3%) outcomes. The good perceived work ability was not affected by the full- or part-time schedule of employment. Conversely, blue-collar workers showed a significantly lower WAI score compared to white-collar workers, although the scores remained in a good range (35.5 ± 6.6 vs. 39.7 ± 4.4, *p* = 0.021). Aging (β –0.15, 95%CI [−0.25–−0.05], *p* = 0.002) and an employment length greater than 15 years (β –3.4, 95%CI [−6.7–0.13], *p* = 0.027) could predict a reduction in work ability. Furthermore, a better quality of life, in all its sub-domains, was demonstrated as predictive for enhanced work ability (Table 4). In regards to sickness absence, 41 (42%), 16 (16%), 7 (7%), and 2 (2%) workers reported to have been absent for <10, 10–24, 25–99, and ≥100 days, respectively, while 32 workers (33%) referred to have not been absent at all.

## 4. Discussion

This study represents the first attempt to evaluate the work function of CF patients according to a comprehensive approach that aimed to verify the employment status, type of occupation performed, and perceived work ability of affected subjects. Understanding the possible influence that individual, clinical or workplace factors can have on CF occupational outcomes may provide guidance to achieve targeted strategies for the successful management of the disease. 

Concerning employment status, half of our investigated population retained a full- or part-time job at the time of the survey. Such a rate was in line with the European percentages of 46–65% reported in the UK, Belgian and French CF populations in the 2009–2014 period [8]. Interestingly, our percentage was lower than the 72.9% reported in 2020 for the general Italian population, aged 35–44 years, but comparable to the 51.9% reported in the Campania region [18], supporting the idea that CF does not function as a limitation to patients’ employability. Unfortunately, in our study, no comparison could be made with an equivalent group of healthy matched individuals. In the employed subgroup, a significantly higher percentage of males was present (65%), reflecting the different employment rate registered for males (61.7%) compared to female subjects (35.9%) in the Campania region in 2020 [18]. This may be due to the generally greater involvement of women in household chores, but also to the higher morbidity suffered by CF women, which may prevent workforce participation [19,20]. Moreover, the unemployed subjects were significantly younger than the employed subjects, although this finding may be biased by the inclusion of students in this category. In line with these results, at both the national and regional level, the number of employed subjects generally increased from 25 to 54 years old [18]. However, it cannot be excluded that, in our population, older patients may be more confidential with their disease, and more effective in retaining jobs with respect to CF management. Additionally, the younger age at CF diagnosis determined in the unemployed group may have influenced their educational achievement, due to school absence because of illness, as well as their vocational attainment and career development. This could also be confirmed by the significantly different educational distribution found between the two groups. Higher levels of education could be a key factor in successful professional insertion, as these could allow patients to achieve tertiary, less physically demanding positions, more easily adaptable to their health conditions. This is in line with the national occupational data for the 35–44 age range in 2020. In fact, it has been reported that employment rates increase from 42.8 and 60.1% for primary and secondary school, respectively; 76% for high school diploma; 86% for degree and post-graduate degree. A comparable trend was also described for the south of Italy, although specific results for the Campania region are not available [18]. 

Concerning CF severity features, we could demonstrate that only an increase in the FVC was positively correlated with employment. No other clinical aspects demonstrated such a predictive role, supporting the need to define more sensitive “medical or psychological indicators” that are useful to follow up the success of the management of the disease, with respect to clinical outcomes, but also of personal and professional achievements. In this view, further studies should investigate, in depth, the impact of different genetic mutations, e.g., homozygosity or heterozygosity in F508del, as the most common CFTR worldwide mutation and responsible for different disease phenotypes, may have on employment status and occupational limitations. Previous data on CF students with the G551D mutation, treated with novel genetic CFTR modulator therapy, showed a significantly lower loss in school productivity and activity impairments compared to those patients, with the F508del mutation, who were managed using standard of care [21]. Although this aspect was not addressed in our study, these results support the importance of defining the role that therapeutic innovation, e.g., potentiator usage, may exert on patients’ work function, with respect to traditional approaches. 

From a psychological perspective, when the CF quality of life was explored, the employed subjects showed significantly better scores in some specific sub-domains of the CFQR, such as those assessing the physical component, role functioning, body image, and treatment burden. The better outcomes in these domains were positively associated with the possibility of being employed and achieving a better work ability. 

In this regard, the vast majority of the employed subjects reported a generally good work ability, with significant differences only identified between the white- and blue-collar occupations. This can support the idea that when employed in adequate occupational positions, CF workers may not be limited at all. Aging and a longer length of employment (>15 years) were predictors for a reduction in work ability, while better outcomes in quality of life were reported to have a beneficial impact. This further underlines the importance of a holistic approach for the management of a “person affected by CF”, considering the interplay of several aspects of the patients’ life in determining his/her physical, mental, and social well-being. This finding may regard the social/familiar support that may result in the high resilience of CF patients, with positive coping strategies, in turn promoting workforce participation (a greater percentage of unemployed patients were single). However, it cannot be excluded that having and keeping a job, causing feelings of self-worth and self-esteem, may have a positive effect on social skills and familiar fulfillment. Comparable considerations may be extended to lifestyle habits, such as sport activities and smoking, which may all contribute to CF health, and personal and professional achievements.

## 5. Conclusions

Although our study is limited by its single-center design and by the enrollment of only outpatients, we, nevertheless, investigated a great number of CF patients, accounting for more than two-thirds of the population within the adult CF Centre of our University Hospital. Additionally, we employed questionnaires specifically validated for CF, or widely employed in occupational health research, i.e., to assess the work ability, according to a methodology that has not been used in the CF literature before. Further research is necessary; however, our results may be useful to inform interdisciplinary CF healthcare management, including the assessment of work function, and to define formal career counseling, job guidance related to CF, as well as discussions on job-related issues, to support the personal, social and professional lives of patients.

## Figures and Tables

**Table 1:** Characteristics of the study participants and differences between working and non-working subjects. Data are presented as frequency (percentages) for categorical data and as M ± SD for continuous data. *p*-values referring to working vs. non-working subjects are computed with chi-square or Fisher test as appropriate for categorical data or with Student’s *t* test or Mann–Whitney U test as appropriate for continuous data. Significant values are marked in bold.

	Study Population (N = 196)	Non-Workers N(%) N = 98	Workers N(%) N = 98	*p*-Value
Age (Mean ± SD)	33.4 ± 10.6	30 ± 10	37 ± 10	**<0.001**
Sex N (%)				**<0.001**
Female	92 (47%)	58 (59%)	34 (35%)	
Male	104 (53%)	40 (41%)	64 (65%)	
BMI (Mean ± SD)	24.2 ± 4.2	24.1 ± 4.5	24.3 ± 3.9	0.736
Age at CF diagnosis (Mean ± SD)	12.7 ± 16.2	9 ± 13	17 ± 18	**<0.001**
Sport N (%)				0.070
Yes	121 (62%)	67 (68%)	54 (55%)	
No	43 (22%)	15 (15%)	28 (29%)	
In the past	32 (16%)	16 (16%)	16 (16%)	
Smoker N (%)				**<0.001**
Yes	20 (10%)	3 (3.1%)	17 (17%)	
No	160 (82%)	90 (92%)	70 (71%)	
In the past	16 (8%)	5 (5.1%)	11 (11%)	
Educational level N (%)				**0.039**
Primary/Secondary school	38 (19%)	21 (21%)	17 (17%)	
High school diploma/Enrolled in university	113 (58%)	62 (63%)	51 (52%)	
Degree/Post-graduate degree	45 (23%)	15 (15%)	30 (31%)	
Marital status N(%)				**0.005**
Single	113 (58%)	67 (69%)	46 (47%)	
In a relationship	77 (39%)	29 (29%)	48 (49%)	
Separated/Divorced	6 (3%)	2 (2%)	4 (4%)	
Children N (%)				0.215
Yes	40 (20%)	16 (16%)	24 (24%)	
No	156 (80%)	82 (84%)	74 (76%)	
Lung transplant recipient N (%)				-
Yes	-	-	-	
No	196 (100%)	98 (100%)	98 (100%)	
Spirometric parameters (Mean ±DS)				
FEV_1_ L	2.6 ± 1.2	2.45 ± 1.05	2.78 ± 1.27	0.051
FEV_1_ %	76 ± 30	74 ± 29	78 ± 30	0.283
FVC L	3.5 ± 1.3	3.26 ± 1.13	3.75 ± 1.33	** 0.006 **
FVC %	86 ± 23	84 ± 22	89 ± 24	0.124
FEV_1_/FVC	72 ± 13	73 ± 13	72 ± 13	0.323
Hospital admission in the last year N (%)				0.805
Yes	18 (9%)	10 (10%)	8 (8.2%)	
No	178 (91%)	88 (90%)	90 (92%)	
Oxygen therapy in the last year N (%)				0.282
Yes	15 (8%)	10 (10%)	5 (5.1%)	
No	181 (92%)	88 (90%)	93 (95%)	
IV antibiotic courses in the last year N (%)				0.552
Yes	30 (15%)	17 (17%)	13 (13%)	
No	166 (85%)	81 (83%)	85 (87%)	
Cough in the previous 15 days N (%)				0.064
Yes	45 (23%)	24 (24%)	21 (21%)	
No	151 (77%)	74 (76%)	77 (79%)	
Cystic Fibrosis Questionnaire—Revised (CFQ-R) subdomains				
Physical functioning	65 ± 25	62 ± 25	69 ± 26	**0.036**
Vitality	65 ± 20	64 ± 21	66 ± 18	0.430
Emotional functioning	75 ± 19	72 ± 20	77 ± 18	0.068
Eating problems	92 ± 17	90 ± 20	94 ± 14	0.092
Treatment Burden	58 ± 21	54 ± 17	62 ± 23	**0.009**
Health Perceptions	68 ± 19	67 ± 20	69 ± 19	0.622
Social functioning	69 ± 15	70 ± 16	69 ± 15	0.505
Body image	79 ± 20	75 ± 20	83 ± 19	**0.009**
Role functioning	80 ± 21	76 ± 23	83 ± 18	**0.018**
Weight	82 ± 29	82 ± 29	83 ± 28	0.803
Respiratory symptoms	78 ± 18	76 ± 19	80 ± 17	0.144
Digestive symptoms	75 ± 20	74 ± 20	76 ± 21	0.505
Work ability index score	39 ± 5.1	-	39 ± 5.1	-

BMI, body mass index; FEV1, forced expiratory volume in the first second; FVC, forced vital capacity.

**Table 2:** Differences between part-time and full-time workers. Data are presented as frequency (percentages) for categorical data and as M ± SD for continuous data. *p*-values are computed with chi-square or Fisher test as appropriate for categorical data or with Student’s *t* test or Mann–Whitney U test as appropriate for continuous data. Significant values are marked in bold.

	Part-Time N = 33	Full-Time N = 65	*p*-Value
Age (Mean ± SD)	34 ± 11	39 ± 10	0.057
Sex N (%)			**0.007**
Female	18 (55%)	16 (25%)	
Male	15 (45%)	49 (75%)	
BMI (Mean ± SD)	23.9 ± 4.3	24.5 ± 3.7	0.499
Age at CF diagnosis (Mean ± SD)	13 ± 19	19 ± 17	0.193
Sport N (%)			**0.019**
Yes	17 (52%)	37 (57%)	
No	6 (18%)	22 (34%)	
In the past	10 (30%)	6 (9.2%)	
Smoker N (%)			0.519
Yes	4 (12%)	13 (20%)	
No	24 (73%)	46 (71%)	
In the past	5 (15%)	6 (9.2%)	
Educational level N (%)			0.435
Primary/Secondary school	8 (24%)	9 (14%)	
High school diploma/Enrolled in university	16 (48%)	35 (54%)	
Degree/Post-graduate degree	9 (27%)	21 (32%)	
Marital status N(%)			0.565
Single	17 (52%)	29 (45%)	
In a relationship	14 (42%)	34 (52%)	
Separated/Divorced	2 (6.1%)	2 (3.1%)	
Children N (%)			>0.999
Yes	8 (24%)	16 (25%)	
No	25 (76%)	49 (75%)	
Lung transplant recipient N (%)			-
Yes	0 (0%)	0 (0%)	
No	33 (100%)	65 (100%)	
Spirometric parameters (Mean ±DS)			
FEV_1_ L	2.58 ± 1.23	2.87 ± 1.29	0.284
FEV_1_ %	76 ± 31	79 ± 30	0.643
FVC L	3.53 ± 1.41	3.86 ± 1.29	0.265
FVC %	88 ± 27	89 ± 22	0.861
FEV_1_/FVC	71 ± 12	72 ± 14	0.843
Hospital admission in the last year N (%)			0.436
Yes	29 (88%)	61 (94%)	
No	4 (12%)	4 (6.2%)	
Oxygen therapy in the last year N (%)			0.331
Yes	30 (91%)	63 (97%)	
No	3 (9.1%)	2 (3.1%)	
IV antibiotic courses in the last year N (%)			0.352
Yes	27 (82%)	58 (89%)	
No	6 (18%)	7 (11%)	
Cough in the previous 15 days N (%)			0.859
Yes	6 (18%)	15 (23%)	
No	27 (82%)	50 (77%)	
Cystic Fibrosis Questionnaire-Revised (CFQ-R) subdomains			
Physical functioning	67 ± 24	70 ± 27	0.500
Vitality	67 ± 17	65 ± 19	0.562
Emotional functioning	77 ± 18	77 ± 18	0.949
Eating problems	92 ± 17	95 ± 11	0.382
Treatment Burden	58 ± 20	63 ± 25	0.282
Health Perceptions	71 ± 16	68 ± 20	0.422
Social functioning	70 ± 13	68 ± 16	0.459
Body image	79 ± 20	84 ± 19	0.213
Role functioning	82 ± 16	84 ± 19	0.703
Weight	77 ± 34	86 ± 24	0.186
Respiratory symptoms	77 ± 17	82 ± 17	0.208
Digestive symptoms	69 ± 21	80 ± 20	**0.019**
Work ability Index score	38.9 ± 4.1	39.0 ± 5.6	0.976

BMI, body mass index; FEV1, forced expiratory volume in the first second; FVC, forced vital capacity.

**Table 3:** Productive field of employment and occupational activities performed by CF employed subjects.

Industrial Classification (ISIC)	N (%)
A. Agriculture, forestry and fishing	1 (1)
B. Mining and quarrying	-
C. Manufacturing	7 (7)
D. Electricity, gas, steam and air conditioning supply	3 (3)
E. Water supply, sewerage, waste management and remediation activities	-
F. Construction	6 (6)
G. Wholesale and retail trade; repair of motor vehicles and motorcycles	16 (16)
H. Transportation and storage	5 (5)
I. Accommodation and food service activities	7 (7)
J. Information and communication	2 (2)
K. Financial and insurance activities	4 (4)
L. Real estate activities	-
M. Professional, scientific and technical activities	1 (1)
N. Administrative and support service activities	9 (9)
O. Public administration and defence; compulsory social security	8 (8)
P. Education	3 (3)
Q. Human health and social work activities	13 (13)
R. Arts, entertainment and recreation	4 (4)
S. Other service activities	9 (9)
T. Activities of households as employers; undifferentiated good- and service-producing activities of households for own use	-
U. Activities of extraterritorial organizations and bodies	-
**Occupation Classification (ISCO-08)**	**n (%)**
1—Managers	8 (8)
2—Professionals	14(14)
3—Technicians and associate professionals	15 (15)
4—Clerical support workers	21 (21)
5—Service and sales workers	23 (23)
6—Skilled agricultural, forestry and fishery workers	1 (1)
7—Craft and related trades workers	10 (10)
8—Plant and machine operators, and assemblers	4 (4)
9—Elementary occupations	1 (1)
10—Armed forces occupations	1 (1)

ISIC, International Standard Industrial Classification of All Economic Activities; ISCO-08, International Standard Classification of Occupations version 08 established by the International Labour Organization.

**Table 4:** Possible predictive factors for changes in work ability. Significant values are marked in bold.

	All Workers’ WAI Score			
Variable	N	β	(95% CI)	*p*-Value
**Age**	98	−0.15	−0.25, −0.05	**0.002**
**BMI**	98	−0.16	−0.42, 0.10	0.225
**Sex**	98			0.971
Female		—	—	
Male		−0.04	−2.2, 2.1	
**Age at CF diagnosis**	98	−0.06	−0.11, 0.00	0.051
**Sport**				0.073
Yes		0.84	−2.0, 3.7	
No		−1.9	−5.0, 1.3	
In the past		—	—	
**Smoker**	98			0.392
Yes		1.8	−5.7, 2.2	
No	98	0.12	−3.2, 3.4	
In the past		—	—	
**Marital status**	98			**0.018**
Single		—	—	
In a relationship		−2.5	−4.6, −0.52	
Separated/Divorced		−5.0	−10, 0.08	
**Children**	98			0.410
No		—	—	
Yes		−1.0	−3.4, 1.4	
**Type of contract**	76			0.407
Permanent contract		—	—	
Fixed term contract		−0.42	−3.4, 2.6	
Not known		−2.4	−5.9, 1.1	
**Educational level**	98			0.071
Primary/Secondary school		—	—	
High school diploma/Enrolled in university		2.8	0.04, 5.6	
Degree/Post-graduate degree		3.4	0.38, 6.4	
**Employment duration (years)**	98			**0.027**
<1		—	—	
1–5		1.3	−1.4, 4.0	
6–10		0.72	−2.5, 3.9	
11–15		1.7	−2.2, 5.7	
>15		−3.4	−6.7, −0.13	
**Hospital admission last year**	98			0.443
No		—	—	
Yes		−1.5	−5.2, 2.3	
**Oxygen therapy last year**	98			0.217
No		—	—	
Yes		−2.9	−7.5, 1.7	
**IV antibiotic courses last year**	98			0.624
No		—	—	
Yes		−0.75	−3.8, 2.3	
**Cough in the previous 15 days**	98			**0.009**
No		—	—	
Yes		−3.3	−5.7, −0.83	
**Lung function parameters**				
FEV1 L	98	0.67	−0.13, 1.5	0.100
FEV1 %	98	0.02	−0.01, 0.06	0.171
FVC L	98	0.70	−0.06, 1.5	0.070
FVC %	98	0.04	0.00, 0.08	0.063
FEV1/FVC	98	0.01	−0.07, 0.09	0.741
**Cystic Fibrosis Questionnaire—Revised (CFQ-R) subdomains**				
Physical functioning	98	0.11	0.08, 0.15	**<0.001**
Vitality	98	0.10	0.05, 0.15	**<0.001**
Emotional functioning	98	0.09	0.03, 0.14	**0.003**
Eating problems	98	0.03	−0.05, 0.10	0.464
Treatment Burden	98	0.08	0.03, 0.12	**<0.001**
Health Perceptions	98	0.11	0.06, 0.16	**<0.001**
Social functioning	98	0.11	0.04, 0.17	**0.001**
Body image	98	0.08	0.03, 0.13	**0.003**
Role functioning	98	0.14	0.09, 0.19	**<0.001**
Weight	98	0.01	−0.02, 0.05	0.511
Respiratory symptoms	98	0.13	0.08, 0.19	**<0.001**
Digestive symptoms	98	0.05	0.00, 0.10	0.059

BMI, body mass index; FEV1, forced expiratory volume in the first second; FVC, forced vital capacity.

## Supplementary Materials

The following is available online at https://www.mdpi.com/article/10.3390/ijerph182211776/s1: Table S1: possible predictive factors for employment.
