# Supplementary material for: Employment Status and Work Ability in Adults with Cystic Fibrosis
Source: Int J Environ Res Public Health. 2021 Nov 10;18(22):11776. doi: 10.3390/ijerph182211776 (PMC8623674; doi:10.3390/ijerph182211776)
Supplement: Supplementary file 1 [file ijerph-18-11776-s001.zip › ijerph-1450160-supplementary.pdf]

Table S1. Possible predictive factors for employment.

		Worker yes/no		
**Variable**	**N**	**OR**	**95% CI**	**p-value**
**Age**	196	1.07	1.04, 1.11	**<0.001**
**Sex**	196			**<0.001**
Female		—	—	
Male		2.73	1.54, 4.91	
**BMI**	196	1.01	0.95, 1.08	0.734
**Age at CF diagnosis**	196	1.03	1.02, 1.05	**<0.001**
**Sport**	196			0.067
Yes		0.81	0.37, 1.77	
No		1.87	0.74, 4.82	
In the past		—	—	
**Smoker**	196			**<0.001**
Yes		2.58	0.52, 14.7	
No		0.35	0.11, 1.02	
In the past		—	—	
**Educational level**	196			**0.037**
Primary/Secondary school		—	—	
High school diploma/Enrolled in university		1.02	0.49, 2.15	
Degree/Post-graduate degree		2.47	1.02, 6.13	
**Marital status**	195			
Single		—	—	**0.007**
In a relationship		2.50	1.38, 4.58	
Separated/Divorced		2.91	0.55, 21.6	
**Children**	196			0.155
No		—	—	
Yes		1.66	0.83, 3.42	
**Spirometric parameters**				
FEV1 L	196	1.27	1.00, 1.63	**0.050**
FEV1 %	196	1.01	1.00, 1.02	0.280
FVC L	196	1.38	1.09, 1.75	**0.006**
FVC %	196	1.01	1.00, 1.02	0.121
FEV1/FVC	196	0.99	0.97, 1.01	0.320
**Hospital admission last year**	196			0.621
No		—	—	
Yes		0.78	0.29, 2.07	
**Oxygen therapy last year**	196			0.175
No		—	—	
Yes		0.47	0.14, 1.39	
**IV antibiotic courses last year**	196			0.427
No		—	—	
Yes		0.73	0.33, 1.59	
**Cough in the previous 15 days**	196			0.610
No		—	—	
Yes		0.84	0.43, 1.64	
**Cystic Fibrosis Questionnaire-Revised (CFQ-R) subdomains**				
Physical functioning	196	1.01	1.00, 1.02	**0.035**
Vitality	196	1.01	0.99, 1.02	0.427
Emotional functioning	196	1.01	1.00, 1.03	0.065
Eating problems	196	1.01	1.00, 1.03	0.086
Treatment Burden	196	1.02	1.00, 1.03	**0.009**
Health Perceptions	196	1.00	0.99, 1.02	0.619
Social functioning	196	0.99	0.98, 1.01	0.503

Body image	196	1.02	1.00, 1.03	**0.009**
Role functioning	196	1.02	1.00, 1.03	**0.017**
Weight	196	1.00	0.99, 1.01	0.802
Respiratory symptoms	196	1.01	1.00, 1.03	0.141
Digestive symptoms	196	1.00	0.99, 1.02	0.502
